# Different patient versus provider perspectives on living with Cushing’s disease

**DOI:** 10.1007/s11102-024-01381-4

**Published:** 2024-02-05

**Authors:** Amanda Halstrom, I.-Hsin Lin, Andrew Lin, Marc Cohen, Viviane Tabar, Eliza B. Geer

**Affiliations:** 1 Division of Endocrinology, Department of Medicine, Weill Cornell Medicine, New York, NY USA; 2 Department of Epidemiology and Biostatistics, Memorial Sloan Kettering Cancer Center, New York, NY USA; 3 Multidisciplinary Pituitary & Skull Base Tumor Center, Memorial Sloan Kettering Cancer Center, New York, NY USA; 4 Department of Neurology, Memorial Sloan Kettering Cancer Center, New York, NY USA; 5 Department of Neurosurgery, Memorial Sloan Kettering Cancer Center, New York, NY USA; 6 Head and Neck Service, Department of Surgery, Memorial Sloan Kettering Cancer Center, New York, NY USA; 7 Department of Medicine, Memorial Sloan Kettering Cancer Center, New York, NY USA

**Keywords:** Cushing’s, Patient reported outcomes, Quality of life, Virtual education, Pituitary, Rare diseases

## Abstract

**Context:**

Patients with Cushing’s disease (CD) face challenges living with and receiving appropriate care for this rare, chronic condition. Even with successful treatment, many patients experience ongoing symptoms and impaired quality of life (QoL). Different perspectives and expectations between patients and healthcare providers (HCPs) may also impair well-being.

**Objective:**

To examine differences in perspectives on living with CD between patients and HCPs, and to compare care goals and unmet needs.

**Design:**

Memorial Sloan Kettering Pituitary Center established an annual pituitary symposium for pituitary patients and HCPs. Through anonymous pre-program surveys distributed at the 2020 and 2022 symposia, patients and HCPs answered questions related to their own sense, or perception of their patients’ sense, of hope, choice, and loneliness in the context of living with CD.

**Participants:**

From 655 participants over two educational events, 46 patients with CD and 116 HCPs were included. Median age of both groups was 51 years. 78.3% of the patients were female vs. 53.0% of the HCPs.

**Results:**

More patients than HCPs reported they had no choices in their treatment (21.7% vs. 0.9%, P < 0.001). More patients reported feeling alone living with CD than HCPs’ perception of such (60.9% vs. 45.5%, P = 0.08). The most common personal care goal concern for patients was ‘QoL/mental health,’ vs. ‘medical therapies/tumor control’ for HCPs. The most common CD unmet need reported by patients was ‘education/awareness’ vs. ‘medical therapies/tumor control’ for HCPs.

**Conclusions:**

CD patients experience long term symptoms and impaired QoL which may in part be due to a perception of lack of effective treatment options and little hope for improvement. Communicating experiences and care goals may improve long term outcomes for CD patients.

**Supplementary Information:**

The online version contains supplementary material available at 10.1007/s11102-024-01381-4.

## Introduction

Patients with rare diseases face challenges receiving appropriate care. Cushing’s disease (CD), a condition associated with excess endogenous glucocorticoids due to an ACTH-secreting pituitary tumor, is a rare disease, occurring in 0.7 to 2.4 per million per year [1]. Patients with CD are at high risk for metabolic, cardiovascular, and psychiatric disease, in addition to long-term symptom burden and impaired quality of life (QoL), despite adequate treatment [1–3].

A critical aspect of effective patient care is communication and mutual understanding between healthcare provider (HCP) and patient. Patients with pituitary tumors experience significant anxiety associated with their diagnosis, in large part due to difficulties interacting with healthcare systems and limited communication of information [4]. Many pituitary patients express concern regarding the complexity of their care, and satisfaction improves with the delivery of more information by the HCP [4]. Patients with pituitary tumors, and CD specifically, require multidisciplinary care which necessitates effective communication in order to provide the best possible outcomes [5].

Similar to acromegaly patients [6], CD patients’ long-term well-being may be adversely affected by different perspectives and expectations between patients and HCPs, especially after treatment [7]. While HCPs primarily use biochemical data to define successful treatment, patients rely more on their symptoms and ability to regain normal functioning [7]. Despite achieving biochemical remission, CD patient perception of having persistent disease negatively impacts QoL [8]. In addition, 67.5% of Cushing’s syndrome patients report receiving insufficient information from their HCPs regarding the recovery experience after surgery despite the fact that all HCPs report providing this information [9]. Improved communication between HCPs and CD patients is vital to optimizing patients’ QoL and long term outcomes.

Recently there has been a growing emphasis on the use of internet-based platforms for healthcare delivery and education [10]. With the goals of offering HCP and patient education and assessing pituitary patients’ needs, since 2019 the pituitary center at Memorial Sloan Kettering (MSK) has offered annual virtual educational programs for pituitary patients, caregivers, HCPs, and members of the pharmaceutical industry. For the current study, we gathered deidentified information from 2020 to 2022 MSK program participants on CD patients’ and HCPs’ attitudes about CD, related to their sense of hope, choice, and loneliness, through anonymous pre-program surveys. Our specific aims were to: (1) Assess differences in perspectives between patients’ and HCPs’ responses in the pre-program survey; (2) Compare patients’ and HCPs’ perceived care goals and unmet needs.

## Methods

### Educational program enrollment

The MSK program was offered to patients with any type of pituitary tumor as well as HCPs, family members, caregivers, and members of industry. The role of the registrant as a patient, caregiver/family member, HCP, and/or member of industry was determined for all registrants of the virtual programs.

Any patient with a pituitary tumor treated at our center and outside institutions, inclusive of patients at all points along their treatment journey, were invited to register for the virtual education program. HCPs, including endocrinologists, neurosurgeons, otolaryngologists, radiation oncologists, neurologists, ophthalmologists, neuro-oncologists, family medicine and internal medicine physicians, physicians in training and other allied health professionals who treat and manage patients with pituitary diseases were also invited to register. Invitations were sent through email to neuroendocrine experts and endocrinologists, patient support groups on social media, direct messaging to patients with pituitary tumors by their treating physicians and via patient databases, advertisements through endocrine societies, brochure/postcard mailing, and Eventbrite, a virtual platform for live events.

### Study participants

Registrants from MSK virtual programs held on December 5, 2020, (*n* = 328) and April 9, 2022, (*n* = 327) were included in the pool of subjects, among which the qualifying participants were determined.

**Fig. 1:**
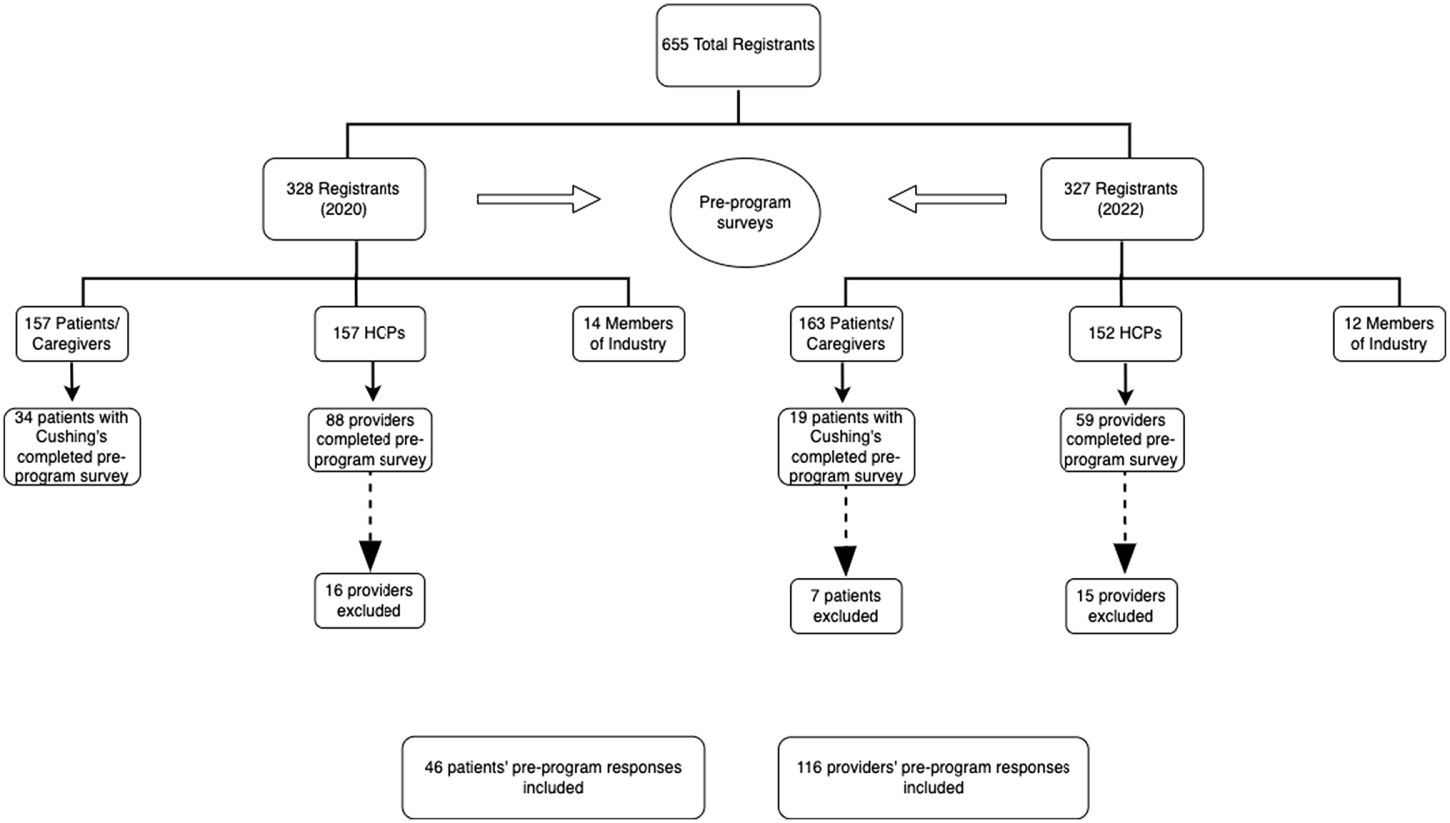
Enrollment flowchart

Of the 655 total registrants from the 2020 and 2022 programs, 320 (48.9%) were patients or caregivers and 309 (47.2%) were HCPs (Fig. 1). Of the 147 providers (88 in 2020 and 59 in 2022) that attended and filled out a pre-program survey 31 were excluded from our analysis. Eight filled out surveys in both 2020 and 2022, 4 were members of industry, 3 did not fill out any responses, and 1 was not in the healthcare field. In addition, 12 providers had at least three fields missing in the survey and 3 had filled out two surveys for the same year, so they were also excluded. A total of 116 providers (72 from 2020 to 44 from 2022) were included in the analysis.

Among the 320 pituitary patients who attended the programs (157 from 2020 to 163 from 2022), 53 identified as ‘patients with Cushing’s’ and submitted surveys (34 participants from 2020 to 19 from 2022). Seven patients were excluded from the 2022 surveys as they had also filled out surveys in 2020, leaving a final group of 46 patients who were included in the analysis.

### Virtual education programs

For each program, there was a single day of live interactive programming, meaning that all participants attended at the same time. The programs were recorded and made available for several weeks as enduring material for registrants on an online website.

After joint sessions in the morning, both programs consisted of two tracks in the afternoon: the ‘provider/clinical track’ and the ‘patient/caregiver track’. During the programs, an ongoing chat reeled through the virtual program which allowed patients to continually ask questions. Faculty experts answered these questions in written responses directly within the chat and/or in spoken responses during one of the live broadcasted Q&A sessions. Additionally, the programs both included panel discussions answering patient questions and moderated patient discussions with invited patient speakers.

### Study procedures

Through anonymous pre-program surveys distributed at the 2020 and 2022 symposia, patients and HCPs answered questions related to their own sense, or perception of their patients’ sense, of hope, choice, and loneliness in the context of living with CD. This survey was developed by a multidisciplinary team and has been reported previously [11]. Demographic and clinical information was also assessed including year of diagnosis, prior treatments, and current medications (for patients) and specialty and practice type (for providers), as shown in Tables 1 and 2. Multiple-choice questions assessing patients’ attitudes toward their disease included possible answers of ‘I have no hope for improvement,’ ‘I have some hope for improvement,’ and ‘I have a lot of hope for improvement;’ and ‘I have no choice in my treatment,’ ‘I have some choices in my treatment,’ and ‘I have many choices in my treatment.’ Patients were also asked to respond ‘TRUE’ or ‘FALSE’ to the following statements: ‘I feel alone living with my Cushing’s,’ ‘Hearing the journeys of other patients helps me better understand my own,’ and ‘I feel anxious about my Cushing’s diagnosis.’

**Table 1:** Patient demographic data

Patients (N = 46)	
Median age (range)	51 (15–75)
Gender	
Female	36 (78%)
Male	10 (22%)
Duration of time with active disease prior to diagnosis (years)	5.26 (0–25)
Duration of time since diagnosis (years)	5.9 (0–33)
Duration of time since surgery (years)	5.64 (0–32)
Number of patients who underwent surgery for Cushing’s (%)	42 (91%)
Number of surgeries (range)	1.17 (0–4)
Number that received radiation (%)	9 (20%)
Duration of time since radiation, if received (years)	6.11
Current Cushing’s therapy	
Metyrapone	5 (11%)
Ketoconazole	4 (9%)
Cabergoline	3 (7%)
Osilodrostat	2 (4%)
Pasireotide, mifepristone, temozolomide	0
Hormone replacement therapy	
Thyroid replacement	17 (37%)
Adrenal replacement	14 (30%)
Estrogen or testosterone replacement	11 (24%)
Growth hormone replacement	5 (11%)

Mean values provided unless otherwise noted

**Table 2:** Provider demographic data

Providers (N = 116)	
Median age (range)	51 (25–86)
Gender	
Female	61 (53%)
Male	54 (47%)
Medical specialty	
Endocrinology	83 (72%)
Neurosurgery	11 (9%)
Nursing	9 (8%)
Oncology	3 (3%)
ENT	2 (2%)
Critical Care	2 (2%)
Other*	6 (5%)
Practice type	
Private practice	19 (16%)
Academic/University Hospital	13 (11%)
Unspecified clinical care	18 (16%)
Unspecified hospital based	18 (16%)
Unspecified	44 (38%)
Other**	4 (3%)

Mean (%) unless otherwise noted

*Includes: Aerospace Medicine, Dermatology, Dietician, Pediatric Endocrinology, Inpatient Acute Care, Pain Medicine

**Includes: Community Hospital, Research Investigator, Government

Multiple-choice questions assessing providers’ attitudes about their patients' Cushing’s included possible answers of ‘I have no hope for their improvement,’ ‘I have some hope for their improvement,’ and ‘I have a lot of hope for their improvement;’ and ‘my patients have no choice in their treatment,’ ‘my patients have some choices in their treatment,’ and ‘my patients have many choices in their treatment.’ Providers were also asked to respond ‘TRUE’ or ‘FALSE’ to the following statements: ‘my patients feel alone living with their Cushing’s,’ ‘Hearing the journeys of other patients helps will help my patients better understand their own,’ and ‘my patients feel anxious about their Cushing’s diagnosis.’

Additionally, patients were surveyed on care goals and unmet needs related to their treatment. Specifically, patients were asked, ‘What are the healthcare outcomes/goals that matter to you the most?’ and ‘What do you think are unmet needs for the diagnosis or treatment of your condition?’ The first question was intended to refer to the patient specifically, while the second question was meant to examine how the condition is treated in general. Survey responses were submitted as free text and subsequently grouped by the authors (AH and EBG) into nine different categories: (a) Quality of life (QoL)/Mental Health; (b) Medical Therapies/Tumor Control; (c) Education/Awareness; (d) Communications/Multidisciplinary Care; (e) Insurance/Access; (f) Fertility; (g) Controlling Comorbidities; (h) Support System and (i) none. Responses could receive multiple designations if applicable. AH coded the free text themes independently, then EBG reviewed each answer and corresponding grouping to confirm accuracy. If there was disagreement or confusion, coding from our prior work [11] was reviewed.

HCPs were also surveyed on care goals and unmet needs related to their patient’s treatment. Providers were asked, ‘What are the healthcare outcomes/goals that matter to you the most?’ and ‘what do you think are unmet needs for the diagnosis or treatment of your patient’s condition?’ The first question was intended to refer to the provider and their goals related to Cushing’s, while the second question was meant to examine how the condition is treated in general. Survey responses were submitted as free text and subsequently grouped by the authors (AH and EBG) into nine different categories: (a) Quality of life (QoL)/Mental Health; (b) Medical Therapies/Tumor Control; (c) Education/Awareness; (d) Communications/Multidisciplinary Care; (e) Insurance/Access; (f) Fertility; (g) Controlling Comorbidities; (h) Support System and (i) none. Responses could receive multiple designations if applicable.

### Statistical analysis

Descriptive statistics were presented as counts and percentages for categorical variables and as medians and interquartile range (IQR) for continuous variables. The Chi-square test or Fisher’s exact test was used to compare gender and survey responses between the CD patient group and the HCP group. All statistical tests were two-tailed, and a P-value of < 0.05 was considered statistically significant. SAS Software® (version 9.4; SAS Institute Inc., Cary, NC) was used for all analyses.

## Results

**Fig. 2:**
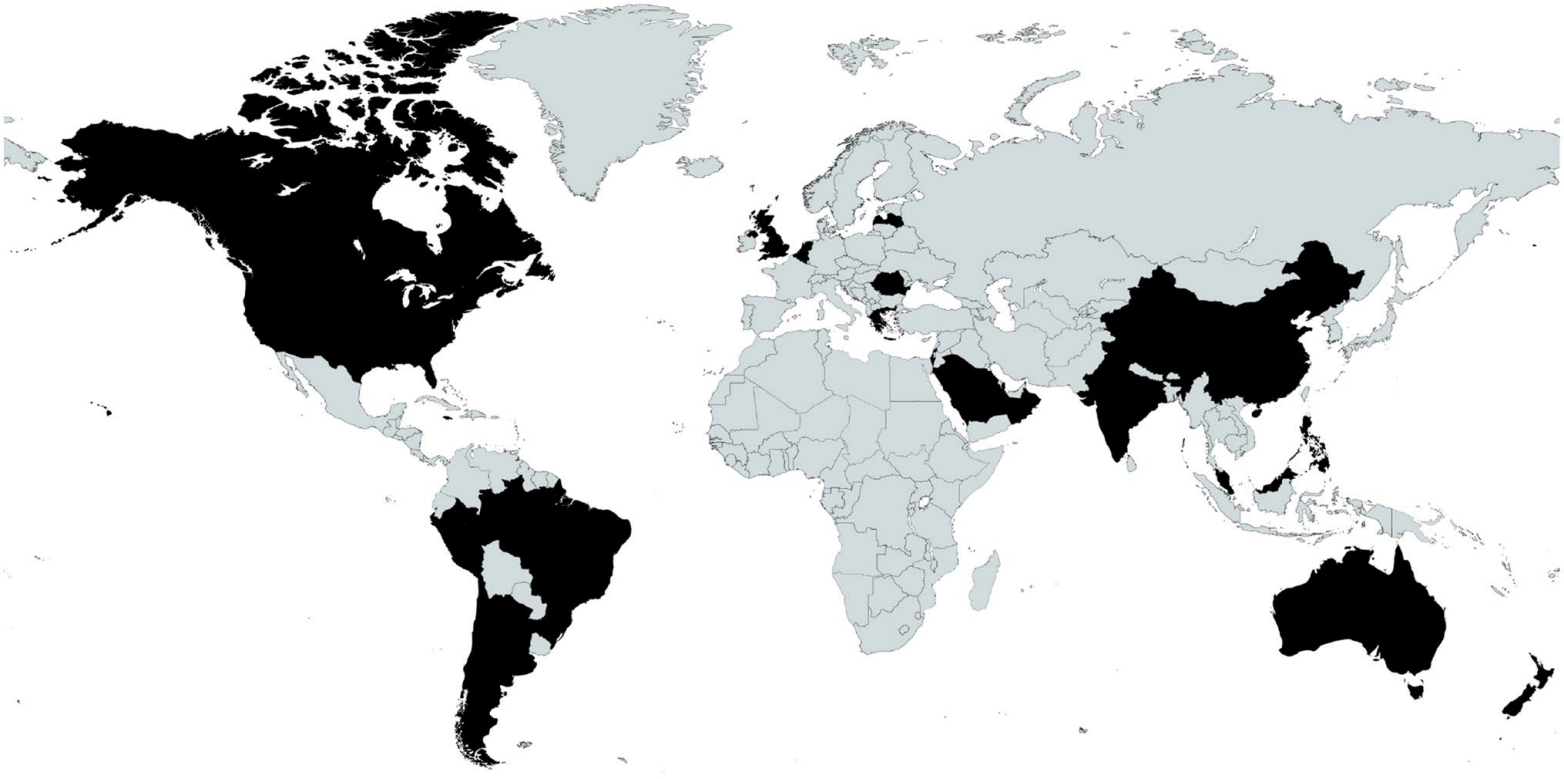
Map of registrant locations. Locations (listed alphabetically): Argentina, Australia, Belgium, Brazil, Canada, Chile, China, Greece, Hong Kong, India, Israel, Jamaica, Latvia, Malaysia, Netherlands, New, Zealand, Oman, Peru, Philippines, Qatar, Romania, Saudi Arabia, Singapore, UK, US

Between the 2020 and 2022 events, there was combined representation from 25 different countries. A map and a full list of the countries is shown in Fig. 2.

From a total of 655 participants over two educational events, 46 patients with CD and 116 HCP caring for CD patients were included in the analysis. The demographic data of the patients and HCPs are outlined in Tables 1 and 2, respectively. Median age of the patients and HCPs was 51 years. 78.3% of the CD group was female vs. 53.0% of the HCP group (P = 0.003).

CD patients ranged from newly diagnosed to being diagnosed 33 years prior. The HCPs who filled out the pre-program surveys were in practice for a mean duration of 18.5 years, with a range from 1 to 54 years.

As shown in Table 1, CD patients had a mean duration of suspected active disease prior to diagnosis of 5.26 years, as defined by onset of CD symptoms until diagnosis, and a mean duration of disease since diagnosis of 5.9 years. 42 (91%) had undergone surgical treatment of their Cushing’s. For those who underwent surgery, the mean number of surgeries was 1.17 (range 0–4). 20% had received pituitary radiation. Overall, 31% of patients were on medical therapy for Cushing’s. Metyrapone was the most used CD therapy (in 11%), followed by ketoconazole (in 9%). Of those requiring pituitary hormone replacement, 34.8% had one pituitary hormone deficiency and 21.7% had multiple hormone deficiencies. Thyroid hormone replacement (37%) and adrenal replacement (30%) were the most common.

As shown in Table 2, the majority of the HCPs were endocrinologists (72%) followed by neurosurgeons (9%) and nurses (8%). There was a total of 9 different specialties represented by the provider group. 16% of the providers worked in private practice, 16% were hospital based, and 16% worked in ‘unspecified clinical care.’ 38% of the providers practice type was ‘unspecified.’

Based on the pre-program survey responses, we identified different attitudes between patients and HCPs in several domains. Table 3 depicts pre-program survey responses from CD patients and HCPs assessing their attitudes about CD. 21.7% of patients reported they had no choices in their treatment, compared to 0.9% of HCPs (P < 0.001). Almost all HCPs (99.1%) reported that CD patients had least some choice in their management. In addition, less than half (45.7%) of patients reported they had a lot of hope for improvement whereas 71.3% of HCPs had a lot of hope for their patients’ improvement. Surprisingly, fewer CD patients reported feeling anxious about their diagnosis compared to HCPs’ perceived patient anxiety (65.2% vs 94.6%, P < 0.001). However, more patients tended to feel more alone living with CD than HCPs’ perception of such (60.9% vs. 45.5%, P = 0.08). Both CD patients and HCPs agreed that hearing the journeys of other CD patients would help patients better understand their own disease (97.8% vs 100%).

**Table 3:** Patient and provider attitudes by pre-program survey

Answers	Patient response (N = 46)	Provider response (N = 116)	P-value
A lot of hope for improvement	21 (45.7%)	82 (71.3%)	*
No choice in the management of CD	10 (21.7%)	1 (0.9%)	** < 0.001**
Feeling alone living with CD	28 (60.9%)	46 (45.5%)	0.08
Feeling anxious living with CD	30 (65.2%)	105 (94.6%)	** < 0.001**
Hearing the journeys of other patients with CD improves understanding	45 (97.8%)	113 (100%)	*

Statistically significant difference in attitudes between providers and patients are given in bold

*Statistical testing not possible due to ‘0-value’ in sample

**Fig. 3:**
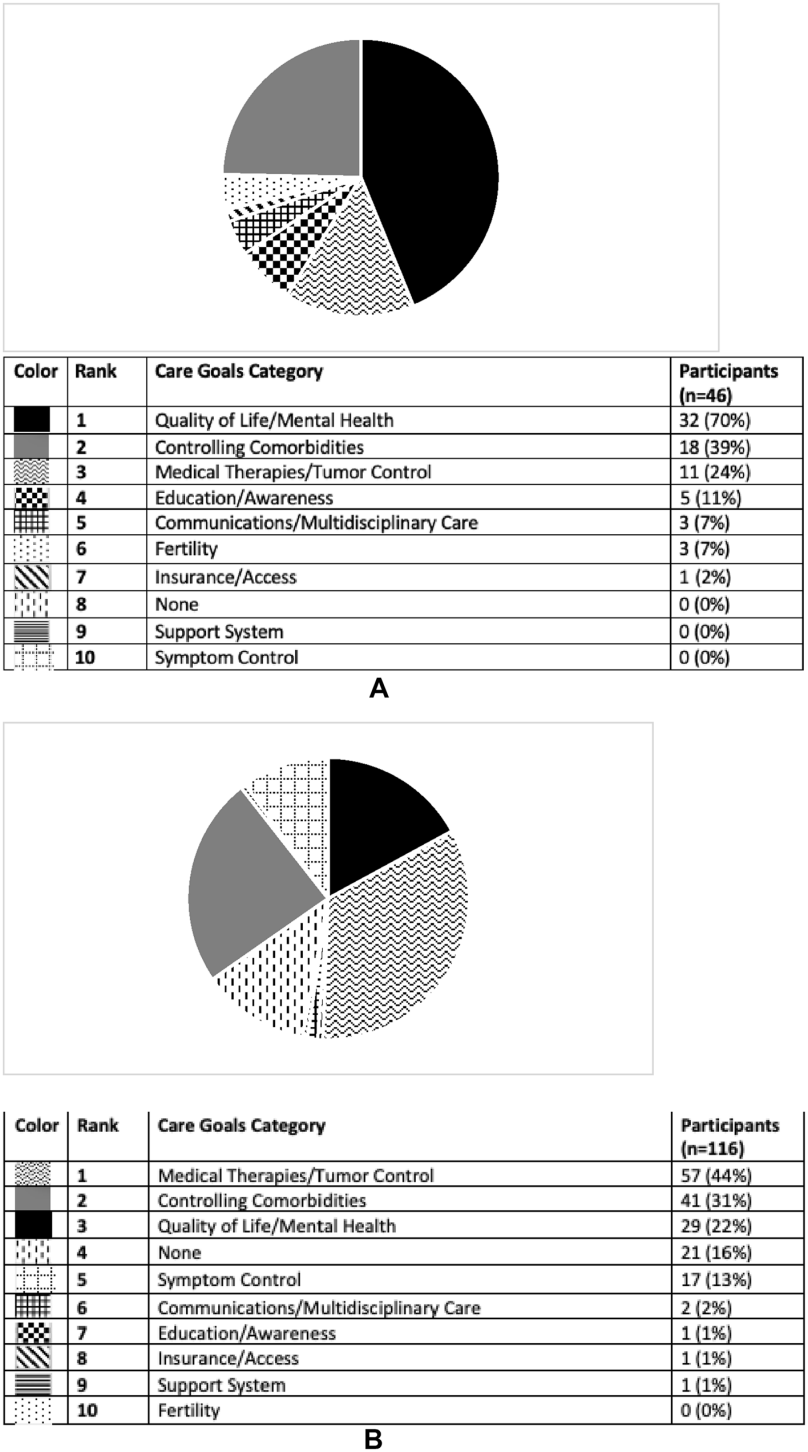
**A** Care goals according to participants with Cushing’s who completed pre-program survey. This pie graph represents the free-text survey response from patients regarding their personal care goals as categorized by topic. **B** Care goals according to providers who completed pre-program survey. This pie graph represents the free-text survey response from providers regarding their personal care goals as categorized by topic

**Fig. 4:**
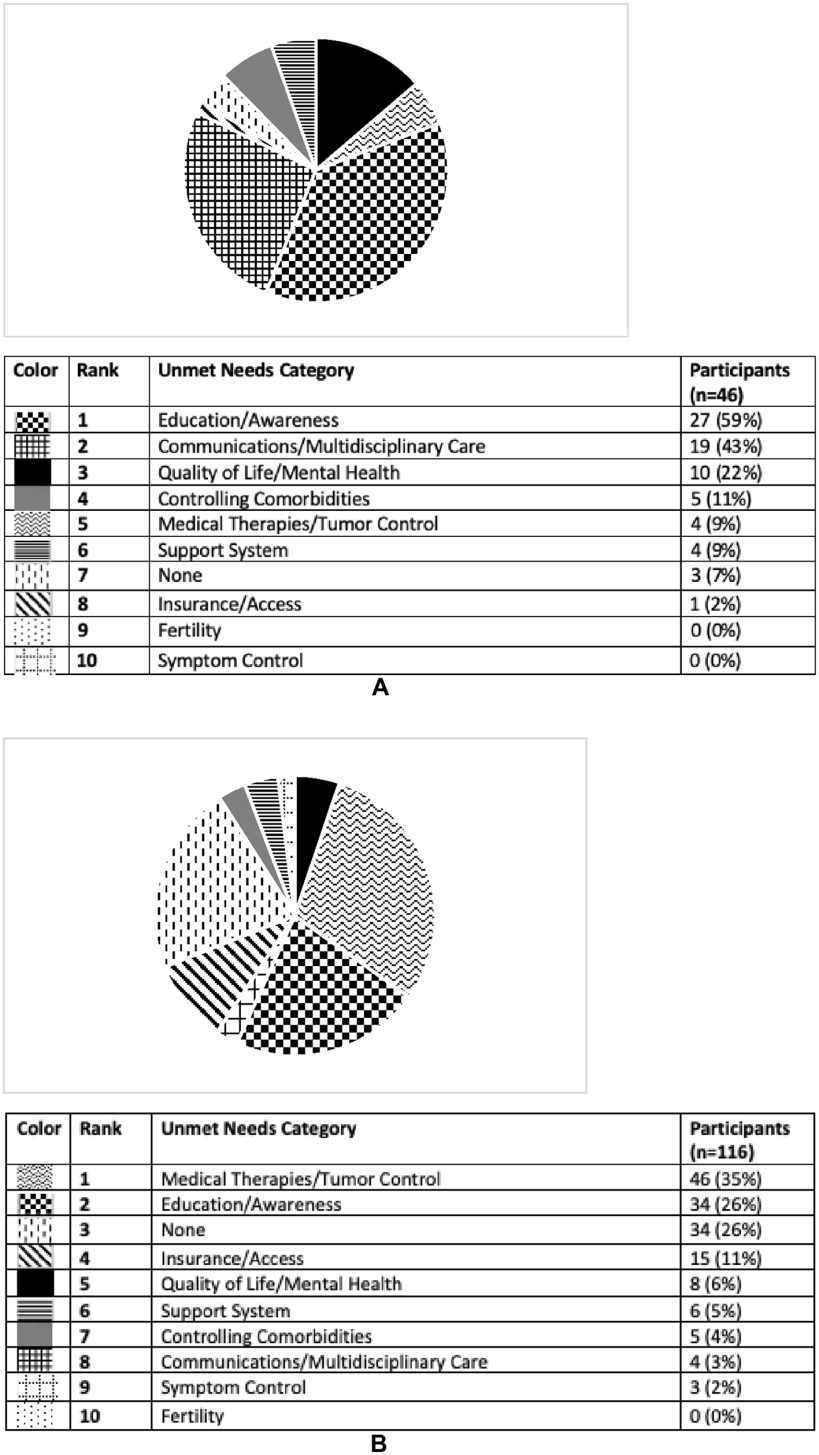
**A** Unmet needs for the field of Cushing’s disease according to participants with Cushing’s who completed pre-program survey. This pie graph represents the free-text survey response from patients regarding unmet needs in Cushing’s as categorized by topic. **B** Unmet needs for the field of Cushing’s disease according to providers who completed pre-program survey. This pie graph represents the free-text survey response from providers regarding unmet needs in Cushing’s as categorized by topic

CD patients and HCPs were also surveyed on their personal care goals and unmet needs, results of which are shown in Figs. 3A, B and 4A, B. The most common personal care goal concern for patients was ‘QoL/mental health’ which was reported by 70%, followed by ‘controlling comorbidities’ (39%) and ‘medical therapies/tumor control’ (24%). HCPs prioritized the same three care goals as patients but ‘medical therapies/tumor control’ was the most common (44%). ‘Controlling comorbidities’ and ‘QoL/mental health’ were the second and third most often HCP reported care goals (31 and 22% respectively). ‘Education/awareness’ was the most common perceived CD unmet need by patients (59%). HCPs reported both ‘medical therapies/tumor control’ and ‘education/awareness’ to be the most common unmet needs (35 and 26%, respectively). Examples of patient and provider responses, and how they were coded, are shown in Supplemental Table 1.

## Discussion

This study examined the differences between patients and HCP-reported perceptions of living with CD. We identified several differences in disease outlook between CD patients and HCPs. We found that more patients than HCPs reported they had no choices in their treatment. Furthermore, less than half of patients reported they had a lot of hope for improvement whereas most (71.3%) of HCPs had a lot of hope for their patients’ improvement. Interestingly, fewer CD patients reported feeling anxious about their diagnosis compared to HCPs’ perceived patient anxiety, although a higher percentage of patients reported feeling alone living with CD compared to the HCPs’ perception of patient loneliness. We also identified HCP and patient differences in reported personal care goals and perceived unmet needs in the field. The most common personal care goal concern for patients was ‘QoL/mental health,’ whereas it was ‘medical therapies/tumor control’ for HCPs. ‘Education/awareness’ was the most commonly perceived unmet need by patients, whereas it was ‘medical therapies/tumor control’ for HCPs.

Our findings support prior work demonstrating a discrepancy between patients and HCPs regarding the need for improved multidisciplinary care [12]. 43% of patients listed ‘communication/multidisciplinary care’ as an unmet need in the field, compared to 3% of providers. Pituitary centers of excellence provide expert multidisciplinary care in the neuroendocrine, neurosurgical, and radiation oncology domains, but often lack expertise in mental and physical health domains salient for CD patients, who suffer from depression, anxiety, myopathy and joint pain. In order to offer comprehensive care, psychiatrists, psychologists, social workers, pain medicine experts, physical therapists, and nutritionists with expertise in CD should be included in the pituitary center multidisciplinary team [13]. Our findings suggest that pituitary centers of excellence should take into account the most important personal care goal reported by CD patients, which is Qol/mental health, and provide expert treatment in this domain.

It is not surprising that Qol/mental health is the personal care goal most reported by CD patients. Prior assessment of acromegaly patients demonstrated the same finding: QoL/mental health was the most common personal care goal concern [11]. While surgical [14] and medical [15–18] treatment of Cushing’s improves QoL, QoL has been shown to remain impaired over time after treatment [19]. Several factors may contribute to long-term Qol impairments, including the presence of persistent disease, imperfect treatment modalities which themselves may be associated with burden and adverse side effects, and persistent comorbidities including depression, anxiety, fatigue, and overweight. Perception of disease status may also play a role in QoL. In surgically remitted CD patients, there may be discordance between biochemical remission and perceived disease status [8]. Specifically, this study found that of those with self-identified persistence of disease, 65% were in fact biochemically remitted. This group had lower QoL scores than the concordant group who self-identified as in remission with biochemical evidence of remission.

CD patients’ outlook on their condition, including their perception of choices and hope for change, has not been previously well described, despite the fact that these perceptions likely inform long term Qol. Patient outlook may be a modifiable target that if addressed, could improve long term patient well-being and outcomes. Aside from continuing progress in the development of new therapies for CD patients which can offer patients more objective choices in their treatment, other modalities should be considered. Prior work has shown that virtual educational programs improve acromegaly patients’ hope for improvement, perception of having choices in their treatment, and sense of loneliness [11]. Educational programs have also been shown to result in improved physical activity and sleep, and reduced pain levels in CS patients [20]. More work is needed to develop effective education programming tailored for CD patients to provide the appropriate support that these patients need.

Difference in HCP and patient disease perceptions may also play a role in Cushing’s patients’ quality of life and outcomes. Among a cohort of patients who underwent surgical resection for Cushing’s, 32.4% reported not receiving information from their doctors about the recovery experience, despite the fact that all physicians surveyed reported giving information about the recovery process [9]. Furthermore, 16.1% of patients in this cohort reported that not enough medical professionals were familiar with the symptoms of Cushing’s. Recovery time was also reported to be longer by patients than providers [9]. Similarly, discordance was found between acromegaly patients and HCPs regarding reported severity of symptoms, with patients more frequently reporting symptoms as severe compared to HCPs, and many patients reporting symptoms which were not reported by HCPs [6]. Improving communication between HCP and patients may positively affect CD patient outlook and QoL.

We identified a similar disparity between CD patients and HCP regarding care goals and unmet needs. 70% of patients surveyed considered QoL/mental health to be a top care goal, but only 22% of provider shared this goal. 59% of patients reported education/awareness as an unmet need, compared to 26% of HCPs. These findings support data shown by Acre et al. in which Cushing’s patients report a lack of symptom recognition by their providers [9]. HCPs should be aware that their patients may have different treatment priorities.

Our finding that more HCPs reported patient anxiety living with CD compared to patients themselves needs further exploration. This could reflect inadequate communication between HCP and patient, or skewed HCP perceptions of CD. This, and other findings in our study should be viewed in light of the small cohort, and as such, needs confirmation in larger cohorts and more in-depth symptom assessments. Additional limitations of our study include lack of paired patient-HCP responses as the HCPs included were not providing care for this specific CD cohort. Since this was a pituitary educational forum, likely most or all patients who identified as having Cushing’s had CD. However, our survey did not specify the type of surgery patients underwent or the etiology of their Cushing’s. Additionally, we used multidisciplinary team agreed upon measures and not validated assessments. Further work should consider validating a tool to assess patient-provider discordances. Our findings may also be confounded by selection bias, given that the patients participating in our virtual education programs are more likely to be under the care of experts in the field and may not represent the attitudes of all patients living with CD. Finally, the included HCPs were representatives from a range of specialties with different levels of experience taking care of patients with CD which may also affect their responses.

Our findings highlight the importance of understanding CD patients’ outlook and perspective in their condition, and that they may differ from their HCP. More than half of CD patients did not have a lot of hope for improvement and reported feeling alone, and many patients felt they had no choices in their treatment. QOL/mental health was the most commonly reported care goal for patients, which was not the case for HCPs. Comprehensive multidisciplinary care for CD patients should include mental health professionals with expertise in CD. Regular open communication between HCPs and CD patients will help bridge perception differences and facilitate personalized care, which will ultimately improve long-term outcomes for CD patients.

### Supplementary Information

Below is the link to the electronic supplementary material.

Supplementary file1 (DOCX 15 kb)

## Data Availability

The data that support the findings of this study are available from the authors upon request.
