# Supplementary material for: Different patient versus provider perspectives on living with Cushing’s disease
Source: Pituitary. 2024 Feb 5;27(2):141–50. doi: 10.1007/s11102-024-01381-4 (PMC11009766; doi:10.1007/s11102-024-01381-4)
Supplement: Supplementary file 1 — Supplementary file1 (DOCX 15 kb) [file 11102_2024_1381_MOESM1_ESM.docx]

**Supplemental Table 1: Examples of Coded Patient and Provider Responses to Open-Ended Questions**

|  | **Patient Response** | **Provider Response** |
| --- | --- | --- |
| **Quality of Life/Mental Health** | I want to be able to function somewhat like I used to and be able to work and enjoy life. Right now I can barely make it to work 30 hours and you can just forget a social life. | Mental health |
| **Medical Therapies/Tumor Control** | Controlling with medication, managing long term effects. | Multiple medications which are not fully effective. |
| **Education/Awareness** | I'd like more education as a patient and I wish there was more awareness in the medical profession and public at large. | Swift and accurate diagnosis, appropriate treatment, sufficient after care and follow up, support for mental health |
| **Insurance/Access** | More testing for certain hormones and overall health. I think my blood testing should be more frequent. I see an endocrinologist but not one that specialized in pituitary diseases. | Easier testing |
| **Controlling Comorbidities** | Losing the weight and not living in chronic pain and fatigue and sinusitis and always labs are off. | Cushingoid facies and body weight |
| **Communications/Multidisciplinary Care** | There is a disconnect between my Cushing's care team (like my endocrinologist) and my mental health care team (my psychiatrist and therapist). I wish they were more integrated. | Speech, physical and occupational therapy |
| **Support System** | Emotional support (therapy, support groups, etc.) | Patient support |
| **Symptom Control** | N/A | Managing symptoms can be challenging |
| **Fertility** | I want to return to a normal life and understand the potential for me to be able to become pregnant in the future | N/A |
